# Brain-derived Neurotrophic Factor Regulates Energy Expenditure Through the Central Nervous System in Obese Diabetic Mice

**DOI:** 10.1155/EDR.2001.201

**Published:** 2001

**Authors:** Takeshi Nonomura, Atsushi Tsuchida, Michiko Ono-Kishino, Tsutomu Nakagawa, Mutsuo Taiji, Hiroshi Noguchia

**Affiliations:** 1 Sumitomo Pharmaceuticals Co. Ltd. Discovery Research Laboratories II 3-1-98 Kasugadenaka Konohana-ku Osaka 554-0022 Japan; 2 Sumitomo Pharmaceuticals Co. Ltd. Business Development and Licensing Office 3-11 Kandasurugadai Chiyoda-ku Tokyo 101-8319 Japan

## Abstract

It has been previously demonstrated that brain-derived neurotrophic factor (BDNF) regulates glucose metabolism and energy expenditure in rodent diabetic models such as C57BL/KsJ-lepr^db^/lepr^db^ (*db/db*) mice. Central administration of BDNF has been found to reduce blood glucose in *db/db* mice, suggesting that BDNF acts through the central nervous system. In the present study we have expanded these investigations to explore the effect of central administration of BDNF on energy metabolism. Intracerebroventricular administration of BDNF lowered blood glucose and increased pancreatic insulin content of *db/db* mice compared with vehicle-treated pellet pair-fed *db/db* mice. While body temperatures of the pellet pair-fed *db/db* mice given vehicle were reduced because of restricted food supply in this pair-feeding condition, BDNF treatment remarkably alleviated the reduction of body temperature suggesting the enhancement of thermogenesis. BDNF enhanced norepinephrine turnover and increased uncoupling protein-1 mRNA expression in the interscapular brown adipose tissue. Our evidence indicates that BDNF activates the sympathetic nervous system *via* the central nervous system and regulates energy expenditure in obese diabetic animals.


*Int. Jnl. Experimental Diab. Res.*, Vol. 2, pp. 201–209
Reprints available directly from the publisher
Photocopying permitted by license only

© 2001 OPA (Overseas Publishers Association) N.V.
Published by license under
the Harwood Academic Publishers imprint,
part of Gordon and Breach Publishing,
a member of the Taylor & Francis Group.
Printed in the U.S.A.

### Brain-derived Neurotrophic Factor Regulates Energy Expenditure Through the Central Nervous System in Obese Diabetic Mice

TAKESHI NONOMURA[a], ATSUSHI TSUCHIDA[a], MICHIKO ONO-KISHINO[a], TSUTOMU NAKAGAWA[a], MUTSUO TAIJI[a,*] and HIROSHI NOGUCHI[a,b]

[a]*Sumitomo Pharmaceuticals Co. Ltd., Discovery Research Laboratories II, 3-1-98 Kasugadenaka, Konohana-ku, Osaka 554-0022, Japan;* [b]*Sumitomo Pharmaceuticals Co. Ltd., Business Development and Licensing Office, 3-11 Kandasurugadai, Chiyoda-ku, Tokyo 101-8319, Japan*

*(Received 2 April 2001; Revised 29 June 2001; In final form 30 July 2001)*

**It has been previously demonstrated that brain-derived neurotrophic factor (BDNF) regulates glucose metabolism and energy expenditure in rodent diabetic models such as C57BL/KsJ-lepr$^{db}$/lepr$^{db}$ (*db/db*) mice. Central administration of BDNF has been found to reduce blood glucose in *db/db* mice, suggesting that BDNF acts through the central nervous system. In the present study we have expanded these investigations to explore the effect of central administration of BDNF on energy metabolism. Intracerebroventricular administration of BDNF lowered blood glucose and increased pancreatic insulin content of *db/db* mice compared with vehicle-treated pellet pair-fed *db/db* mice. While body temperatures of the pellet pair-fed *db/db* mice given vehicle were reduced because of restricted food supply in this pair-feeding condition, BDNF treatment remarkably alleviated the reduction of body temperature suggesting the enhancement of thermogenesis. BDNF enhanced norepinephrine turnover and increased uncoupling protein-1 mRNA expression in the interscapular brown adipose tissue. Our evidence indicates that BDNF activates the sympathetic nervous system *via* the central nervous system and regulates energy expenditure in obese diabetic animals.**

*Keywords*: Neurotrophic factor; Intracerebroventricular administration; Energy expenditure; Glucose metabolism; Norepinephrine turnover

*Abbreviations*: BDNF, brain-derived neurotrophic factor; NE, norepinephrine; UCP-1, uncoupling protein-1; BAT, brown adipose tissue

### INTRODUCTION

Brain-derived neurotrophic factor (BDNF), a member of the neurotrophin family, has been widely demonstrated to function in the central and peripheral nervous systems and motor neurons in the fetus and in adulthood.[1,2,3,4,5,6] BDNF is known to regulate neural development and regeneration, promote neurite extension and maintain neuronal survival.[7,8,9,10,11] In addition to those diverse roles of BDNF in the nervous system, we have discovered that BDNF plays important roles in the endocrine system and regulates glucose metabolism.[12]

We have shown that systemic administration of BDNF improves glucose metabolism in obese diabetic C57BL/KsJ-lepr$^{db}$/lepr$^{db}$ (*db/db*) mice.[12,13] Although BDNF also suppresses food intake in such hyperphagic obese mice, we developed a novel apparatus to pair-feed vehicle-treated control mice precisely to BDNF-treated mice and demonstrated that BDNF has a major hypoglycemic effect independent of appetite.[14] We have clarified the unique profile of peripheral BDNF administration in regulating glucose metabolism: (1) BDNF enhances insulin sensitivity

*Corresponding author. Tel.: 81-6-6466-5299, Fax: 81-6-6466-5491, e-mail: taiji@sumitomopharm.co.jp and ameliorates insulin resistance; (2) the hypoglycemic effect of BDNF lasts long after the cessation of treatment; and (3) insulin content in pancreas is increased and in histological observations, insulin-positive pancreatic beta cells are regranulated by BDNF administration.[13,14,15] Interestingly, in addition to its efficacy on glucose metabolism, BDNF also prevents the reduction of body temperature in the *db/db* mice deprived of food supply.[14] This finding indicates that BDNF ameliorates the impaired energy balance in diabetic mice. However, the mechanism by which BDNF regulates glucose metabolism and energy expenditure still remains unclear. We have not yet obtained any evidence to show the direct effects of BDNF on glucose metabolism in cultured cells from peripheral tissues such as liver, muscle, and adipose tissue. Under the precise control of food intake by means of our pellet pair-fed apparatus, we have demonstrated that intracerebroventricular administration of BDNF shows the similar anorectic and hypoglycemic effects as seen in peripheral administration in *db/db* mice.[14] We thus hypothesize that BDNF regulates glucose metabolism by acting through the central nervous system. To evaluate this hypothesis we have analyzed the effects of intracerebroventricular BDNF administration on energy expenditure in the present study. We explore the action of BDNF in regulating thermogenesis and demonstrate the involvement of the sympathetic nervous system in this process.

### MATERIALS AND METHODS

### Animals

Male C57BL/KsJ-*db/db* mice were obtained from Clea Japan Inc. (Tokyo, Japan). Mice were singly housed and the treatments started at 10–12 weeks of age. Animals were given food (CE-2, Clea Japan Inc.) and water *ad libitum* except for the pair-feeding experiment. Pellet pair-fed mice were housed in the synchronized pellet pair-feeding apparatus (Sumitomo Pharmaceuticals and Osaka Micro Systems, Osaka, Japan). Pair-feeding experiments were performed basically as described in our previous study.[14] The supply of pellets to the BDNF-treated mice was not limited, but the supply of pellets to the vehicle-treated mice was limited to the number of those consumed by the BDNF-treated mice. All animal experiments were done according to the guidelines of the Sumitomo Pharmaceuticals Committee on Animal Research.

### Intracerebroventricular Administration of BDNF

Human recombinant BDNF (N-terminal methionine-free, Regeneron Pharmaceuticals, Tarrytown, NY) was administered using artificial cerebrospinal fluid (aCSF; 0.166 g/L $CaCl_2$, 7.014 g/L NaCl, 0.298 g/L KCl, 0.203 g/L $MgCl_2/6H_2O$ and 2.10 g/L $NaHCO_3$) as a vehicle for intracerebroventricular administration. Mice were anesthetized with diethyl ether, and fifteen micrograms of BDNF (3 μl/mouse) were injected through a Hamilton syringe into the lateral cerebral ventricle according to the following coordinates: 1.0 mm lateral to the bregma and 3.0 mm ventral to the skull surface. For the pellet-pair feed experiment, mice received a total of five injections, alternating sides of the head for each injection, with the injections being given every other day (three on one side of the head and two on the other side). Both sides of injection placement were verified by injecting Evans Blue in the same manner at the end of the experiment.

### Measurement of Blood Glucose and Insulin

Blood samples were collected from tail vein, and blood glucose was measured by the GLUCOSE CII-TEST WAKO (Mutarotase-glucose oxidase method, Wako Chemical, Osaka, Japan). Plasma insulin concentrations were measured by ELISA (Levis-insulin-mouse; Shibayagi, Gunma, Japan). At the end of the treatment, the whole pancreas was resected from each mouse and divided into splenic and duodenal regions. Splenic regions were weighed, minced, and homogenized in acid-ethanol solution (75% ethanol, 23.5%

distilled water, 1.5% conc. HCl). After overnight incubation at 4°C the suspensions were centrifuged, and the supernatants were collected and assayed for insulin content.

### Body Temperature and Thermographic Imaging Analysis

Body temperature was measured using an electron thermistor (Model BAT-12, Physitemp, Clifton, NJ) equipped with rectal probe (RET-3, Physitemp, Clifton, NJ). Skin temperature was imaged by thermography (TVS-8000MkII, Abionics, Tokyo, Japan) after shaving the back hair.

### Measurement of Norepinephrine (NE) Turnover

The effect of BDNF on norepinephrine turnover was assessed using a slightly modified version of the method previously reported by Collins.[16] *db/db* mice received intracerebroventricular administration of either BDNF (15 μg/mouse) or vehicle at the beginning of a dark cycle, and then food was removed. Two hours after BDNF or vehicle treatment, α-methyl-*p*-tyrosine methyl ester (250 mg/kg, Sigma, St. Louise, MO), an inhibitor of tyrosine hydroxylase, was intraperitoneally injected to block *de novo* catecholamine synthesis. The mice were decapitated two hours after α-MT injection and the interscapular brown adipose tissue (BAT) was immediately dissected, weighed and then frozen in liquid nitrogen. The BAT was homogenized with 0.1 N perchloric acid containing 5 mM EDTA. Homogenates were filtrated through a 0.22 μm mesh membrane to remove debris. Norepinephrine content in homogenates was measured using an HPLC system (LC-10A, Shimadzu Instrumentation, Kyoto, Japan) equipped with a column (CA-5DS, Eicom, Kyoto, Japan).

### Northern Blot Analysis

*db/db* mice were intracerebroventricularly injected with either BDNF or vehicle at the beginning of a dark cycle and then food was removed. Animals were sacrificed 4 hours after BDNF (15 μg/mouse) or vehicle treatment; interscapular BAT was excised and frozen immediately. RNA was prepared from the tissues with Trizol (Gibco BRL Life Technologies, Rockville, MD, USA) using the manufacturer's protocol. Yield and purity of RNA were determined by spectrophotometric absorption analysis at 260/280 nm. 3 μg of total RNA was electrophoresed in a 1% agarose gel containing formaldehyde and then transferred to GT probe membranes (Bio-Rad Laboratories, Hercules, CA, USA). A 1071-base pair rat uncoupling protein-1 (UCP1) probe (nucleotides 84-1154 in Genebank accession no. M11814) was obtained by reverse transcriptase-polymerase chain reaction (RT-PCR) from rat BAT RNA using primers 5′-CCA CAG GAA TTC GAA GTT GAG AGT TCG GTA and 5′-CCC AGC TCT AGA GCC CAG CAT AGG AGC CCA as reported previously.[17] A 349-base pair mouse β-actin probe (nucleotides 728-1076 in Genebank accession no. M12481) was obtained by RT-PCR from mouse liver RNA using primers 5′-TGG AAT CCT GTG GCA TCC ATG AAA C and 5′-TAA AAC GCA GCT CAG TAA CAG TCC G. All probes were verified by sequencing. Probes were randomly labeled using a *Bca*Best labeling kit (Takara, Ohtsu, Japan) with [α-$^{32}$P]-deoxy CTP (Amersham Pharmacia Biotech, Buckinghamshire, England). Hybridization was carried out at 65°C in 0.25 M sodium phosphate (pH 7.2)/7% SDS, and blots were washed twice with 20 mM sodium phosphate (pH 7.2)/5% SDS and then with 20 mM sodium phosphate (pH 7.2)/1% SDS. Hybridization signals were quantified using a bio-imaging analyzer BAS2000 (Fuji Photo Film, Tokyo, Japan).

### Statistical Analysis

All data are presented as means ± SD. The statistical calculations were performed using SAS software (SAS Institute, Cary, NC), and differences between individual groups were analyzed by the Student's *t*-test, the Dunnett's test or Jonckheere-Terpstra test. $P < 0.05$ was considered statistically significant.

### RESULTS

### Effect of Intracerebroventricular Administration of BDNF on Glucose Metabolism

15 μg BDNF per mouse or the same volume (3 μl/mouse) of vehicle solution was administered intracerebroventricularly to *db/db* mice every other day 5 times. After intracerebroventricular BDNF administration, the food intake of *db/db* mice decreased as shown in Figure 1A. Food intake of the vehicle-treated pellet pair-fed *db/db* mice was very well synchronized to the BDNF-treated mice. Compared with such vehicle-treated control mice, the repetitive intracerebroventricular administration of BDNF significantly lowered blood glucose concentrations in *db/db* mice (Fig. 1B). There was no significant difference in body weight between BDNF-treated and the pellet pair-fed mice (Fig. 1C). To study the dose-dependency of repetitive intracerebroventricular administration, three different doses (0.15, 1.5 and 15 μg/mouse) of BDNF were injected every other day to *db/db* mice, respectively. BDNF was found to be dose-dependently effective in lowering blood glucose concentration and reducing food intake of *db/db* mice by Jonckheere-Terpstra test (blood glucose; P = 0.002, food intake; P = 0.009). 15 μg/mouse of BDNF significantly reduced food intake and lowered blood glucose concentration (Figs. 2A, B).

In addition to blood glucose, we next analyzed the effect of intracerebroventricular administration of BDNF on plasma insulin levels. As shown in Table I, plasma insulin concentrations of both

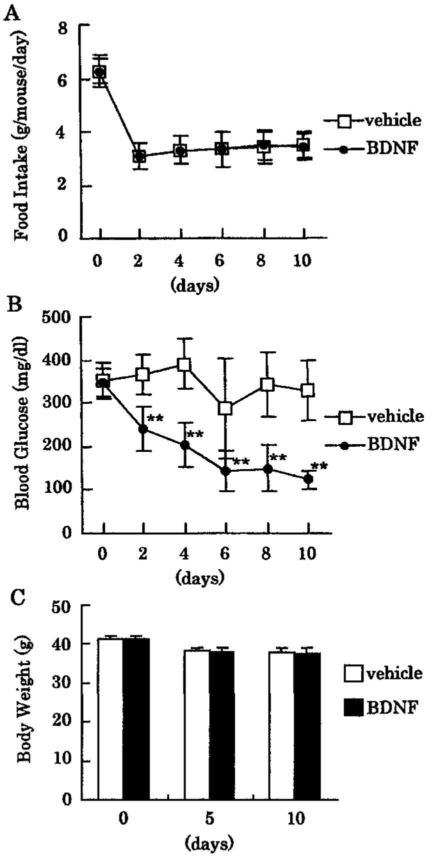

FIGURE 1 Effects of intracerebroventricular BDNF administration on food intake (A), blood glucose concentration (B) and body weight (C) in *db/db* mice. BDNF (15 μg/mouse) or vehicle was administered on alternate days to *db/db* mice housed in the pellet pair-feeding apparatus. Data are presented as mean ± SD (n = 9). **P < 0.01 *vs.* vehicle by Student's *t*-test.

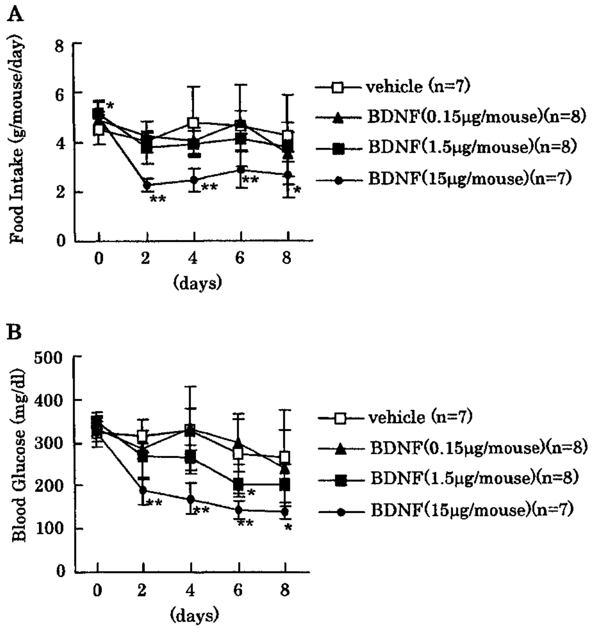

FIGURE 2 Dose-response effects of intracerebroventricular BDNF administration on food intake (A) and blood glucose concentration (B) in *db/db* mice. BDNF (0.15, 1.5, 15 μg/mouse) or vehicle was administered on alternate days to *ad libitum*-fed *db/db* mice. Data are presented as mean ± SD (n = 7 or 8). *P < 0.05, **P < 0.01 *vs.* vehicle by Dunnett's test. BDNF was found to be dose-dependently effective in lowering blood glucose concentration and reducing food intake of *db/db* mice by Jonckheere-Terpstra test (blood glucose; P = 0.002, food intake; P = 0.009).

TABLE I Effect of intracerebroventricular BDNF administration on plasma insulin concentration and pancreatic insulin content in *db/db* mice. BDNF (15 μg/shot) or vehicle was administered on alternate days for a total of five injections to *db/db* mice housed in the pellet pair-feeding apparatus. Plasma insulin concentration was measured at Days 0 (baseline) and 10. Pancreases were removed from the mice at Day 10 and the pancreatic insulin content was measured. Data are presented as mean ± SD (n = 9)

| Day | 1 | 10 |
|---|---|---|
| Plasma insulin concentration [ng/ml] | | |
| Pair-feed + vehicle | 45.1 ± 20.9 | 30.4 ± 16.1 |
| BDNF | 49.0 ± 11.6 | 16.3 ± 12.2 |
| Pancreatic insulin content [ng/mg tissue] | | |
| Pair-feed + vehicle | N.A. | 92.9 ± 47.3 |
| BDNF | N.A. | 606.8 ± 161.4** |

**P < 0.01 *vs.* vehicle by Student's *t*-test.

the BDNF-treated *db/db* mice and pellet pair-fed *db/db* mice decreased during the experimental period. However, the plasma insulin concentration of BDNF-treated *db/db* mice tended to be lower than that of the pellet pair-fed mice after repetitive intracerebroventricular administrations. Since we have previously found that subcutaneous administration of BDNF increases pancreatic insulin contents of *db/db* mice, we then analyzed pancreatic insulin content after repetitive intracerebroventricular administration of BDNF. The pancreatic insulin content of BDNF-treated mice was found to be approximately 6-fold higher than that of the pellet pair-fed mice (Tab. I). These findings suggest that intracerebroventricular administration as well as subcutaneous administration of BDNF regulates glucose metabolism in a similar fashion.

### Effect of Intracerebroventricular Administration of BDNF on Body Temperature

To verify our hypothesis that BDNF regulates glucose metabolism by acting through the brain, we analyzed the effect of intracerebroventricular BDNF administration on the rectal temperature of *db/db* mice in this study. Compared with *ad libitum*-fed *db/db* mice (approximately 37–38°C), the rectal temperature of the vehicle-treated pellet pair-fed *db/db* mice was lower, probably due to the reduced food intake that was synchronized with BDNF-treated mice (Tab. II). The rectal temperature of the BDNF-treated *db/db* mice at Days 1 and 10 was significantly higher than the vehicle-treated pellet pair-fed *db/db* mice and almost comparable to *ad libitum*-fed mice in spite of a reduced food intake that was approximately the same as the pair-fed mice. We then examined the skin temperature of these *db/db* mice by thermography imaging analysis (Fig. 3). Whereas the skin temperature of a typical vehicle-treated pellet pair-fed mouse was lower than an *ad libitum*-fed mouse, the skin temperature of the paired BDNF-treated mouse recovered. A relatively higher temperature was observed in the interscapular region of the BDNF-treated mouse suggesting enhancement of thermogenesis in the brown adipose tissue (BAT).

TABLE II Rectal temperature of *db/db* mice with intracerebroventricular BDNF administration. BDNF (15 μg/shot) or vehicle was administered on alternate days for a total of five injections to *db/db* mice housed in the pellet pair-feeding apparatus. Rectal temperatures were measured at the next day after the first injection (Day 1) and two days after the last (fifth) injection (Day 10). Data are presented as mean ± SD (n = 9)

| Day | 1 | 10 |
|---|---|---|
| Rectal temperature (°C) | | |
| Pair-feed + vehicle | 34.6 ± 1.8 | 35.0 ± 1.1 |
| BDNF | 37.0 ± 0.4** | 37.2 ± 1.2** |

**P < 0.01 *vs.* vehicle by Student's *t*-test.

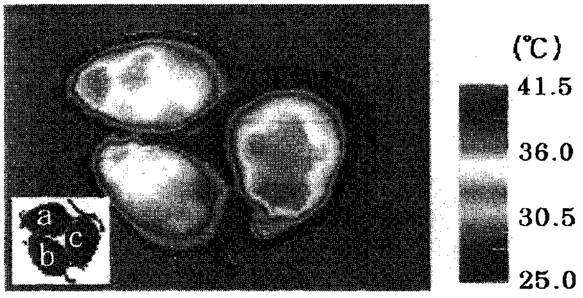

FIGURE 3 Thermographic imaging analysis of the back skin temperature of *db/db* mice after intracerebroventricular BDNF administration. Thermographic imaging analysis of a BDNF (15 μg/mouse, on alternate days)-treated mouse (a), a vehicle-treated mouse pair-fed to the BDNF-treated mouse (b), and a vehicle-treated mouse fed *ad libitum* (c). Analysis was performed at Day 10.

### Enhancement of Norepinephrine Turnover in *db/db* Mice Treated with BDNF

In order to explore the effect of BDNF on sympathetic nerve activity, we examined NE utilization (*i.e.*, NE turnover) in BAT of a BDNF-treated *db/db* mouse. To assess NE turnover, NE contents in BAT were measured after blocking catecholamine synthesis with administration of α-methyl-*p*-tyrosine (α-MT), a tyrosine hydroxylase inhibitor. Two hours prior to α-MT administration, *db/db* mice received a single intracerebroventricular administration of either BDNF or vehicle. After administration of α-MT there was a decrease in the NE contents of the BAT in *db/db* mice that received vehicle intracerebroventricularly, indicating a blockage of catecholamine synthesis. Compared with such control animals, intracerebroventricular administration of BDNF elicited a larger reduction in NE contents in interscapular BAT, indicating enhancement of NE turnover (Fig. 4).

### Effects of a Single Intracerebroventricular Administration of BDNF on mRNA Expression of Uncoupling Protein-1

To study the action mechanism by which BDNF enhances energy expenditure in greater detail we examined the effect of intracerebroventricular administration of BDNF on the expression of uncoupling protein (UCP)-1 gene in BAT. *db/db* mice were intracerebroventricularly administrated with either BDNF (15 μg/mouse) or vehicle followed by food removal. Four hours after BDNF or vehicle administration, total RNA was prepared from BAT and subjected to Northern blot analysis using UCP-1 cDNA as a probe. As shown in Figure 5, a single intracerebroventricular administration of BDNF increased UCP-1 mRNA in BAT by 1.5-fold.

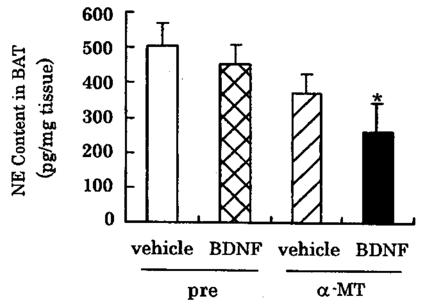

FIGURE 4 NE turnover in *db/db* mice treated with BDNF. Following a single intracerebroventricular administration of BDNF (15 μg/mouse), the tyrosine hydroxylase inhibitor α-methyl-*p*-tyrosine (α-MT) was intraperitoneally injected. NE contents were measured 2hr after injecting the blocking reagent. NE turnover was determined from the decrease in NE content after blockage of catecholamine biosynthesis. Data are presented as mean ± SD (n = 6 or 8). *P < 0.05 *vs.* vehicle by Student's *t*-test.

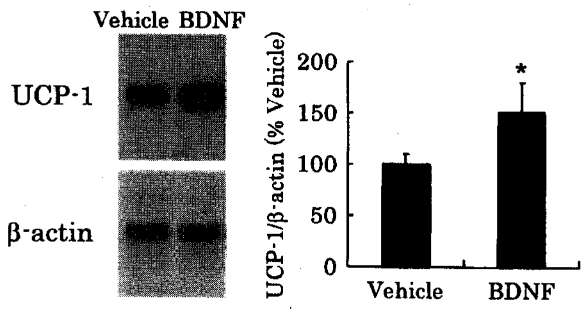

FIGURE 5 Effects of a single intracerebroventricular administration of BDNF on mRNA expression of uncoupling protein-1 in brown adipose tissue.

BDNF (15 μg/mouse) or vehicle was administered intracerebroventricularly to *db/db* mice and then fasting was started. Four hours after BDNF or vehicle administration, the interscapular brown adipose tissue was dissected and total RNA was isolated. Northern blot analysis (UCP1 and β-actin probes) of the total RNA (3 μg) was then performed. Data are shown as means ± SD (n = 4). *P < 0.05 *vs.* vehicle by Student's *t*-test. The left panels show representative blots.

### DISCUSSION

We have previously shown that peripheral subcutaneous administration of BDNF lowered food intake and blood glucose concentration of diabetic *db/db* mice with accompanying obesity and hyperinsulinemia.[12,13] We have also demonstrated the hypoglycemic effect of BDNF on *db/db* mice even under strict pellet pair-feeding conditions using our novel apparatus[14] which indicates that blood glucose is actually being lowered by BDNF apart from the hypoglycemic effect ascribed to hypophagia. Since BDNF does not lower blood glucose levels of normal rodents and streptozotocin-treated rodent models, it is unlikely that

BDNF enhances insulin secretion from the pancreas.[12,14] In streptozotocin-treated mice, we have found concomitant administration of BDNF with insulin enhances the acute hypoglycemic effect of insulin.[14] These data suggest that peripheral subcutaneous administration of BDNF enhances insulin sensitivity or ameliorates insulin resistance or both in peripheral tissues.

In our studies so far with cultured adipocyte and myotubule cell lines we have observed no direct action of BDNF on insulin stimulated 2-deoxyglucose uptake (unpublished data), although peripheral tissues such as adipose tissue and muscle are involved in insulin-dependent glucose metabolism. Moreover, it was reported elsewhere that intracerebroventricular infusion of BDNF suppresses food intake and body weight gain but does not affect blood glucose level in normoglycemic (Long-Evans) rats.[18] Therefore, we investigated the effect of central administration of BDNF on glucose metabolism in hyperglycemic animals. Our present study clearly demonstrated that central BDNF administration reduces blood glucose and also increases pancreatic insulin contents in obese hyperglycemic *db/db* mice under strict pellet pair-feeding conditions. In comparison with subcutaneous administration, a much lower dose (approximately 1/100) of BDNF was found to be effective with central administration. These results indicate that BDNF regulates glucose metabolism and maybe pancreatic function through the central nervous system.

Previously, we have found that subcutaneous administration of BDNF raised rectal and skin temperatures in *db/db* mice,[14] indicating the regulatory role of BDNF on energy metabolism. In the present study we were also able to reproduce these effects of BDNF through central administration. It is well known that the sympathetic nervous system is involved in regulating thermogenesis and maintaining body temperature in mammals.[19] In this study, we demonstrated that central administration of BDNF rapidly enhances NE turnover in thermogenic brown adipose tissue (BAT) of *db/db* mice. This is consistent with the present thermographic data in which skin temperature increased in the interscapular region that abundantly contains BAT. BAT is a major source of non-shivering thermogenesis in rodents[20] and the thermogenic ability of BAT is thought to be due to UCP-1.[21] Intracerebroventricular administration of BDNF also rapidly increased UCP-1 mRNA expression in BAT of *db/db* mice demonstrating the central regulation of BDNF in energy expenditure. Taken together, the above indicates that BDNF regulates energy metabolism through the central nervous system and the autonomic nervous system.

It is well known that the blood brain barrier restricts the transport of peptides and proteins between the blood and the brain.[22] It has been reported however that BDNF passes through the blood brain barrier by a saturable transport system and quickly enters into brain.[23,24] In our preliminary experiments, subcutaneous treatment of BDNF as well as intracerebroventricular treatment rapidly showed anorexic effect in *db/db* mice (data not shown). Since subcutaneous administration of BDNF ameliorated the energy expenditure in our previous study,[14] BDNF administered even peripherally may rapidly enter the brain and regulate energy metabolism in obese diabetic animals.

The pharmacological profiles of BDNF shown in this study reminded us of leptin, an adipocyte-derived satiety hormone regulating body adiposity by modulating food intake and energy metabolism.[18,25,26] Peripheral administration of leptin stimulates sympathetic nerve activity in interscapular BAT and norepinephrine turnover[16,27] and regulates the expression of UCP1 by modulating the sympathetic nervous system.[28,29] Since the functional form of the leptin receptor (Ob-Rb) is expressed in the hypothalamus, a major site of metabolic regulation by the autonomic nervous system,[30] intracerebroventricular or intrahypothalamic administration of leptin can reproduce most of the effects of peripheral leptin administration.[26,31,32] Leptin administered peripherally may access the hypothalamus *via* receptor-mediated transport, and regulate energy expenditure and food intake primarily by interacting with Ob-Rb in the hypothalamus. Therefore, many of the therapeutic profiles of leptin are very similar to BDNF.

Since the functional full-length form of BDNF receptor, *trk*B is expressed in the hypothalamus,[33,34] it is plausible that BDNF may act *via* the hypothalamic neuronal system. More studies will be needed to clarify the action mechanisms of BDNF in comparison with leptin.

In conclusion, the present study has demonstrated that central administration of BDNF regulates the glucose metabolism and energy expenditure of obese diabetic animals in a similar fashion to peripheral administration. These results further suggest that BDNF modulates sympathetic nerve activity through central regulation in the hypothalamus and affects energy expenditure.

### References

[1] Connor, J. M., Lauterborn, J. C., Yan, Q., Gall, C. M. and Varon, S. (1997). Distribution of BDNF protein and mRNA in the normal adult rat CNS: evidence for anterograde transport. *J. Neurosci.*, **17**, 2295–2313.

[2] Lindsay, R. M., Wiegand, S. J., Altar, C. A. and DiStefano, P. S. (1994). Neurotrophic factors: from molecule to man. *Trends Neurosci.*, **17**, 182–190.

[3] Skup, M. H. (1994). BDNF and NT-3 widen the scope of neurotrophin activity: pharmacological implications. *Acta Neurobiol. Exp.*, **54**, 81–94.

[4] Yamamoto, H. and Gurney, M. E. (1990). Human platelets contain brain-derived neurotrophic factor. *J. Neurosci.*, **10**, 3469–3478.

[5] Yan, Q., Rosenfeld, R. D., Matheson, C. R., Hawkins, N., Lopez, O. T., Bennett, L. and Welcher, A. A. (1997). Expression of brain-derived neurotrophic factor (BDNF) protein in the adult rat central nervous system. *Neuroscience*, **78**, 431–448.

[6] Yuen, E. C., Howe, C. L., Li, Y., Holtzman, D. M. and Mobley, W. C. (1996). Nerve growth factor and the neurotrophic factor hypothesis. *Brain & Development*, **18**, 362–368.

[7] Berninger, B. and Poo, Mm. (1996). Fast actions of neurotrophic factors. *Curr. Opin. Neurobiol.*, **6**, 324–30.

[8] Black, I. B. (1999). Trophic regulation of synaptic plasticity. *J. Neurobiol.*, **41**, 108–118.

[9] Cohen-Cory, S. and Fraser, S. E. (1995). Effects of brain-derived neurotrophic factor on optic axon branching and remodeling *in vivo*. *Nature*, **378**, 192–196.

[10] Korsching, S. (1993). The neurotrophic factor concept: a reexamination. *J. Neurosci.*, **13**, 2739–2748.

[11] McAllister, A. K., Katz, L. C. and Lo, D. C. (1997). Opposing roles for endogenous BDNF and NT-3 in regulating cortical dendritic growth. *Neuron*, **18**, 767–778.

[12] Ono, M., Ichihara, J., Nonomura, T., Itakura, Y., Taiji, M., Nakayama, C. and Noguchi, H. (1997). Brain-derived neurotrophic factor reduces blood glucose level in obese diabetic mice but not in normal mice. *Biochem. Biophys. Res. Comm.*, **238**, 633–637.

[13] Tonra, J. R., Ono, M., Liu, X., Garcia, K., Jackson, C., Yancopoulos, G. D., Wiegand, S. J. and Wong, V. (1999). Brain-derived neurotrophic factor improves blood glucose control and alleviates fasting hyperglycemia in C57BLKS-lepr$^{db}$/lepr$^{db}$ mice. *Diabetes*, **48**, 588–594.

[14] Nakagawa, T., Tsuchida, A., Itakura, Y., Nonomura, T., Ono, M., Hirota, F., Inoue, T., Nakayama, C., Taiji, M. and Noguchi, H. (2000). Brain-derived neurotrophic factor (BDNF) regulates glucose metabolism by energy balance in diabetic mice. *Diabetes*, **49**, 436–444.

[15] Ono, M., Itakura, Y., Nonomura, T., Nakagawa, T., Nakayama, C., Taiji, M. and Noguchi, H. (2000). Intermittent administration of brain-derived neurotrophic factor ameliorates glucose metabolism in obese diabetic mice. *Metabolism*, **49**, 129–133.

[16] Collins, S., Kuhn, C. M., Petro, A. E., Swick, A. G., Chrunyk, B. A. and Surwit, R. S. (1996). Role of leptin in fat regulation. *Nature*, **380**, 677.

[17] Gong, D. W., He, Y., Karas, M. and Reitman, M. (1997). Uncoupling protein-3 is a member of thermogenesis regulated by thyroid hormone, $\beta$ 3-adrenergic agonists, and leptin. *J. Biol. Chem.*, **272**, 24129–24132.

[18] Pelleymounter, M. A., Cullen, M. J. and Wellman, C. L. (1995). Characteristics of BDNF-induced weight loss. *Exp. Neurol.*, **131**, 229–238.

[19] Maickel, R. P., Matussek, N., Stern, D. N. and Brodie, B. (1967). The sympathetic nervous system as a homeostatic mechanism. I. Absolute need for sympathetic nervous function in body temperature maintenance of cold-exposed rats. *J. Pharmacol. Exp. Ther.*, **157**, 103–110.

[20] Himms-Hagen, J. (1985). Brown adipose tissue metabolism and thermogenesis. *Ann. Rev. Nutr.*, **5**, 69–94.

[21] Klingenberg, M. (1990). Mechanism and evolution of the uncoupling protein of brown adipose tissue. *Trends Biochem. Sci.*, **15**, 108–112.

[22] Egleton, R. D. and Davis, T. P. (1997). Bioavailability and transport of peptides and peptide drugs into brain. *Peptides*, **18**, 1431–1439.

[23] Poduslo, J. F. and Curran, G. L. (1996). Permeability at the blood-nerve barrier of the neurotrophic factors: NGF, CNTF, NT-3, BDNF. *Mol. Brain Res.*, **36**, 280–286.

[24] Pan, W., Banks, W. A., Fasold, M. B., Bluth, J. and Kastin, A. J. (1998). Transport of brain-derived neurotrophic factor across the blood-brain barrier. *Neuropharmacology*, **37**, 1553–1561.

[25] Halaas, J. L., Gajiwala, K. S., Maffei, M., Cohen, S. L., Chait, B. T., Rabinowitz, D., Lallone, R. L., Burley, S. K. and Friedman, J. M. (1995). Weight-reducing effects of the plasma protein encoded by the obese gene. *Science*, **269**, 543–546.

[26] Campfield, L. A., Smith, F. J., Guisez, Y., Devos, R. and Burn, P. (1995). Recombinant mouse OB protein: evidence for a peripheral signal linking adiposity and central neural networks. *Science*, **269**, 546–549.

[27] Haynes, W. G., Morgan, D. A., Walsh, S. A., Mark, A. L. and Sivitz, W. I. (1997). Receptor-mediated regional sympathetic nerve activation by leptin. *J. Clin. Invest.*, **100**, 270–278.

[28] Scarpace, P. J. and Matheny M. (1998). Leptin induction of UCP1 gene expression is dependent on sympathetic innervation. *Am. J. Physiol.*, **275**, E259–E264.

[29] Commins, S. P., Marsh, D. J., Thomas, S. A., Watson, P. M., Padgett, M. A., Palmiter, R. and Gettys, T. W. (1999). Norepinephrine is required for leptin effects on gene expression in brown and white adipose tissue. *Endocrinology*, **140**, 4772–4778.

[30] Hopkins, D. and Williams, G. (1997). The Hypothalamus, Neuropeptides and Diabetes. *International Textbook of Diabetes Mellitus*, Second Edition, John Wiley & Sons Ltd.

[31] Satoh, N., Ogawa, Y., Katsuura, G., Hayase, M., Tsuji, T., Imagawa, K., Yoshimasa, Y., Nishi, S., Hosoda, K. and Nakao, K. (1997). The arcuate nucleus as a primary site of satiety effect of leptin in rats. *Neurosci. Lett.*, **224**, 149–152.
[32] Satoh, N., Ogawa, Y., Katsuura, G., Numata, Y., Tsuji, T., Hayase, M., Ebihara, K., Masuzaki, H., Hosoda, K., Yoshimasa, Y. and Nakao, K. (1999). Sympathetic activation of leptin *via* the ventromedial hypothalamus. *Diabetes*, **48**, 1787–1793.
[33] Altar, C. A., Siuciak, J. A., Wright, P., Ip, N. Y., Lindsay, R. M. and Wiegand, S. J. (1994). *In situ* hybridization of *trk*B and *trk*C receptor mRNA in rat forebrain and association with high-affinity binding of [$^{125}$I]BDNF, [$^{125}$I]NT-4/5 and [$^{125}$I]NT-3. *Eur. J. Neurosci.*, **6**, 1389–405.
[34] Merlio, J. P., Ernfors, P., Jaber, M. and Persson, H. (1992). Molecular cloning of rat *trk*C and distribution of the *trk* family in the rat central nervous system. *Neuroscience*, **51**, 513–532.

